# Prospective memory and dementia risk: Examining associations with blood‐based neurodegenerative biomarkers

**DOI:** 10.1002/alz70856_105687

**Published:** 2026-01-07

**Authors:** Erin E. Harrington, Jennifer E. Graham‐Engeland, Jacqueline A. Mogle, Mindy J. Katz, Richard B. Lipton, Martin J. Sliwinski, Orfeu M. Buxton, Conner K Ryan, Henrik Zetterberg, Christopher G. Engeland

**Affiliations:** ^1^ University of Wyoming, Laramie, WY, USA; ^2^ Pennsylvania State University, University Park, PA, USA; ^3^ Clemson University, Clemson, SC, USA; ^4^ Albert Einstein College of Medicine, Bronx, NY, USA; ^5^ Department of Neurology, Albert Einstein College of Medicine, Bronx, NY, USA; ^6^ Pennsylvania State University, State College, PA, USA; ^7^ The Pennsylvania State University, University Park, PA, USA; ^8^ University of Gothenburg, Mölndal, Sweden

## Abstract

**Background:**

Prospective memory (PM; i.e., memory for future actions or events) lapses, such as forgetting to attend an appointment or take medication on time, may be one of the earliest indicators of mild cognitive impairment (MCI) and Alzheimer's Disease and related dementias (ADRD). Yet, limited work has examined links between PM and biomarkers of ADRD such as neurodegenerative biomarkers. Determining such associations may be crucial in early identification of MCI and ADRD risk. The present work addressed this gap by examining self‐reported PM lapses and blood‐based levels of β‐amyloid and tau biomarkers among older adults.

**Method:**

Older adults (*N* = 275, Mage=77.02, 68% female, non‐Hispanic White) enrolled in the Einstein Aging Study completed a two‐week protocol, that included blood draws for biomarker assays of β‐amyloid (Aβ40, Aβ42, Aβ42:Aβ40) and phosphorylated tau (pTau181). Participants reported PM lapses at the end of each day of the two‐week period via study‐provided smartphones. Independent regression analyses examined links between neurodegenerative biomarkers and PM lapses (daily reports averaged across the two weeks) within the full sample and stratified by gender. Covariates included age and educational attainment.

**Result:**

Higher levels of pTau181 were associated with reporting more PM lapses on average across the two weeks (*b* = 0.01, *p* = .005). When examined within gender, this effect appeared to be driven by women: higher levels of pTau181 were associated with reporting more PM lapses among women (*n* = 186, *b* = 0.02, *p* < .001) but not men (*n* = 89, *b* = 0.00, *p* = .678). No significant relationships emerged with β‐amyloid (*p*s>.123).

**Conclusion:**

The present findings indicate that in older adults, PM lapses are related to elevated levels of pTau181 in women by not men. This finding is noteworthy, as markers of pTau181 are detectable in blood in preclinical Alzheimer's disease and increases correlate with risk of disease progression. As such, women experiencing more frequent PM forgetting and elevated levels of pTau181 may be at risk for future pathology. However, longitudinal research is needed to determine whether this relationship persists across time and its association with objective cognitive decline. Continued research in this area may inform early risk detection for MCI and ADRD.

